# A universal computational model for predicting antigenic variants of influenza A virus based on conserved antigenic structures

**DOI:** 10.1038/srep42051

**Published:** 2017-02-06

**Authors:** Yousong Peng, Dayan Wang, Jianhong Wang, Kenli Li, Zhongyang Tan, Yuelong Shu, Taijiao Jiang

**Affiliations:** 1 College of Biology, Hunan University, Changsha, 410082, China; 2 National Institute for Viral Disease Control and Prevention, China CDC, Beijing, 102206, China; 3 College of Computer Science and Electronic Engineering, Hunan University, Changsha, 410082, China; 4 Center of System Medicine, Institute of Basic Medical Sciences, Chinese Academy of Medical Sciences & Peking Union Medical College, Beijing, 100005, China; 5 Suzhou Institute of Systems Medicine, Suzhou, Jiangsu, 215123, China

## Abstract

Rapid determination of the antigenicity of influenza A virus could help identify the antigenic variants in time. Currently, there is a lack of computational models for predicting antigenic variants of some common hemagglutinin (HA) subtypes of influenza A viruses. By means of sequence analysis, we demonstrate here that multiple HA subtypes of influenza A virus undergo similar mutation patterns of HA1 protein (the immunogenic part of HA). Further analysis on the antigenic variation of influenza A virus H1N1, H3N2 and H5N1 showed that the amino acid residues’ contribution to antigenic variation highly differed in these subtypes, while the regional bands, defined based on their distance to the top of HA1, played conserved roles in antigenic variation of these subtypes. Moreover, the computational models for predicting antigenic variants based on regional bands performed much better in the testing HA subtype than those did based on amino acid residues. Therefore, a universal computational model, named PREDAV-FluA, was built based on the regional bands to predict the antigenic variants for all HA subtypes of influenza A viruses. The model achieved an accuracy of 0.77 when tested with avian influenza H9N2 viruses. It may help for rapid identification of antigenic variants in influenza surveillance.

Influenza A viruses have caused much mortality and morbidity to human society^1^. Based on their two surface glycoproteins —hemagglutinin (HA), the main antigen for the virus, and neuraminidase (NA), the influenza A virus could be divided into different subtypes^2^, such as H3N2, H5N1, and so on. So far, 18 HA subtypes and 11 NA subtypes have been reported^2^. Owing to rapid mutations on HA, the influenza virus could change its antigenicity frequently, leading to immune escape^3^. Rapid determination of the influenza antigenicity could help identify the antigenic variants in time.

Traditional methods for determining the antigenicity of influenza viruses mainly include the hemagglutination inhibition (HI) assay and the micro-neutralization assay, which are reported to be lack of consistency^4^. Given the feasibility of rapid and high-quality sequence determination of HAs of influenza viruses in influenza surveillance, sequence-based computational methods for understanding the antigenic properties of influenza viruses have been developed and demonstrated to be helpful in influenza prevention^5^,^6^,^7^,^8^,^9^,^10^,^11^. However, currently methods are only available for predicting antigenicity of the human influenza H1N1, H3N2 and the avian influenza H5N1 viruses. The newly emerging flu viruses keep challenging our public health, but there lack sufficient data to build high-quality model for rapid prediction of their antigenicity from sequences.

Here, by computationally analyzing the sequence mutation pattern on HA1 protein and the HA1 structure similarity of nine representative HA subtypes, we found that these HA subtypes possibly had similar antigenic structures. Then we further analyzed the mechanisms of antigenic variation in influenza H1N1, H3N2 and H5N1 viruses based on amino acid residues and regional bands. Finally, we attempted to build a universal computational model for predicting antigenic variant of all HA subtypes of influenza A viruses based on HA1 protein sequences.

## Results

### The mutation patterns of HA1 protein are similar for HA subtypes of influenza A virus

**Figure 1:**
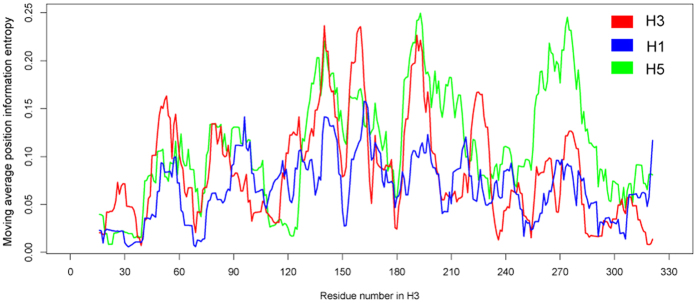
Position-dependent entropy. Moving average position information entropy (MAPIE) was calculated with a window size of 11 for HA1 protein of influenza A virus H1 (blue), H3 (red) and H5 (green). The amino acid positions are numbered according to influenza A virus H3.

The HA1 protein is the immunodominant part of HA, the main antigen of influenza viruses. Consistent with previous reports^12^,^13^, our analyses showed that the nine representative HA subtypes investigated here (H1, H2, H3, H5, H6, H7, H9, H10 and H13) all have very similar HA1 structures (Table S1, Supplementary Information), which was the basis of our hypothesis that all different HA subtypes of influenza A virus have similar antigenic structures. The mutation pattern of HA1 proteins was next analyzed, by calculating the entropy of each amino acid position of the HA1 protein for these nine HA subtypes, which we considered would sufficiently reflect the variation patterns of influenza virus antigens. Figure 1 illustrates the moving average entropy of amino acid positions on HA1 protein of subtypes H1, H3 and H5. For these three subtypes similar patterns of entropy variation of the HA1 residues were observed, with peaks around positions 50, 91, 145, 155, 190 and 275; these positions all belong to the canonical antigenic regions of the protein^14^,^15^. A correlation analysis was performed between the residues’ entropy variation patterns on HA1 protein of nine HA subtypes under investigation. The results (Table S2) showed that all of the HA subtypes had median correlations with other HA subtypes, except for H10. For example, the correlation between the residues’ entropy variation pattern of H1 and other HA subtypes (except for H10) ranged from 0.32 to 0.62 (measured with Pearson Correlation Coefficient (PCC)). In combination, these analyses suggest that the analyzed HA subtypes of influenza A virus most likely contain highly similar antigenic structures.

### The contribution of residues to antigenic variation varies between HA subtypes

We next investigated antigenic variation at the residue level of individual HA subtype. A total of 46, 103 and 26 residues on HA1 were observed to be significantly associated with antigenic variation in influenza A virus H1N1, H3N2 and H5N1, respectively (Table S3). These residues were mostly located in the five canonical antigenic epitopes of the protein^14^,^15^. Surprisingly, there was no correlation between the residues’ contribution to antigenic variation in these subtypes. For example, residue 158 on H3 correlated strongly with antigenic variation, while the corresponding residue hardly correlated with antigenic variation in subtypes H1 or H5. Thus, the contribution of residues to antigenic variation is variable between HA subtypes.

### Regional bands play conserved roles in antigenic variation of influenza A viruses

**Figure 2:**
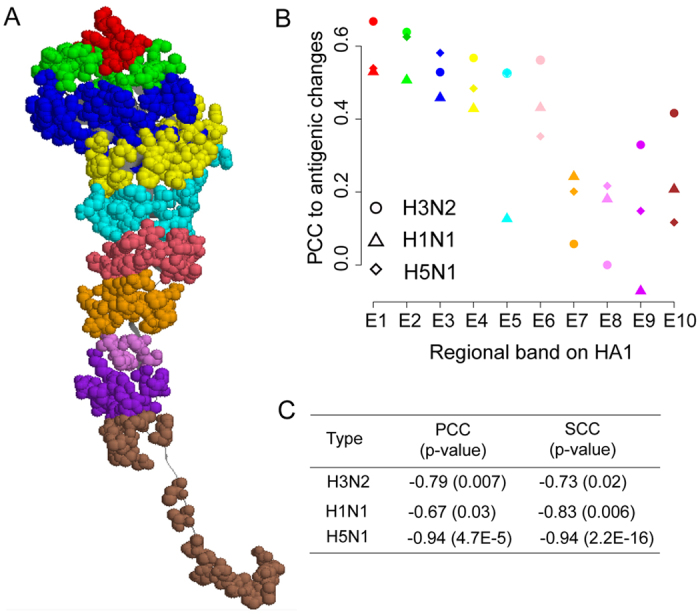
The defined regional bands and their contribution to antigenic variation. (**A**) Visualization of the ten regional bands in HA1 protein defined as described in the methods. Each regional band is differently colored in the 3-D model. (**B**) Pearson correlation coefficients (PCC) between the antigenic variation and the changes in each regional band, for subtype H1N1 (triangles), H3N2 (circles) and H5N1 (diamonds). (**C**) Correlations between the regional band’s contribution to the antigenic variation and their distances from the top of HA1. “SCC”, Spearman correlation coefficient.

We further investigated the mechanisms of antigenic variation at the site level. Ten regional bands (or artificial sites) were defined on the HA1 protein of each HA subtype (Fig. 2A) according to the distances of residues to the top of HA1 (for details see Methods). Nearly all of these bands were observed to contribute significantly to antigenic variation in influenza H1N1, H3N2 and H5N1 viruses, especially for those in the head of HA1 (Fig. 2B and Table S4). The strength of the correlation between the changes of regional bands and the antigenic variation decreased linearly with their distance to the top of HA1 (Fig. 2B): the Spearman Correlation Coefficient (SCC) between them was −0.83 for subtype H1N1, −0.73 for subtype H3N2 and −0.94 for subtype H5N1 (Fig. 2C). This suggests a conserved role of regional bands in the antigenic variation of influenza A virus, independent of the viral subtype.

### Prediction of antigenic variants based on residues and regional bands

Based on our observations that HA subtypes likely share the same antigenic structures and are subject to similar mechanisms generating antigenic variation at the level of regional bands, to build the computational models for predicting antigenic variants of all HA subtypes of influenza A viruses, we firstly investigated whether the computational model based on regional bands trained in one HA subtype could perform well when tested in other HA subtypes. As expected, in most cases, the regional band-based models performed relatively well in the testing subtypes (Table 1), with accuracies greater than 0.75. For example, the model trained in influenza H1N1 viruses achieved an accuracy of 0.75 for subtype H5N1 and 0.78 for subtype H3N2. The latter was close to the accuracy obtained when the model was trained with H3N2. For comparison, we also built models based on the residues. Although the residue-based models performed better than the band-based models in the training subtypes, they performed rather poorly when tested in other subtypes (Table 1 and Table S5).

We further investigated how did the partitioning of the HA1 protein influenced the model of antigenic variant prediction. Besides the models built above, we built models based on a set of five regional bands, which were also used by Lees and his colleagues^6^. This set of bands was also defined according to the distances of residues to the top of the HA1 protein. Interestingly, this kind of models performed similarly with those based on ten regional bands (Table S5). We further built models for which the surface residues on HA1 protein were randomly separated into five bands. As is shown in Table S5, their performances were inferior to the above models based on either ten or five bands, but they performed much better than those did based on residues.

**Figure 3:**
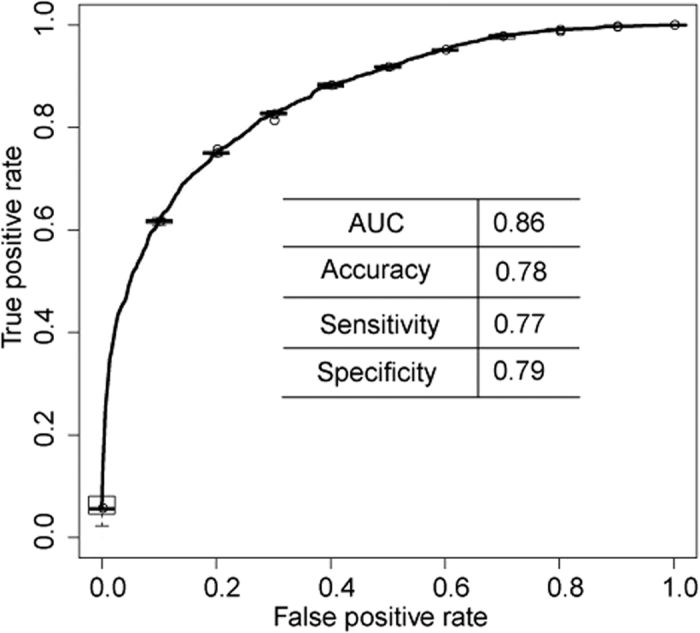
The Receiver Operating Characteristic (ROC) curve of PREDAV-FluA in five-fold cross-validations. The table under the curve summarizes the Area under Curve (AUC) data of the shown curve.

The above pilots suggest that a universal computational model for predicting antigenic variants of all HA subtypes of influenza A virus should preferably be based on regional bands. Therefore, to capture the antigenic variant at the highest possible level of sensitivity, we built a universal computational model called PREDAV-FluA, short for PREDicting Antigenic Variant of inFLUenza A virus, based on the ten regional bands and using the antigenic data of influenza H1N1, H3N2 and H5N1 virus. The model was tested with five-fold cross-validations. Figure 3 shows that this produced a Receiver Operating Characteristic curve with a significant portion of area under curve (AUC) of 0.86 (this value ranges from 0 to 1, with 1 indicating a perfect match with the experimental data). The obtained accuracy was 0.78, with a sensitivity and specificity of 0.77 and 0.79, respectively.

To further demonstrate the value of PREDAV-FluA in predicting antigenic variants of novel subtypes, we collected antigenic data of avian influenza H9N2 viruses, which comprised 118 pairs of sequences with observed antigenic relationship. With these data, the model achieved a moderate accuracy of 0.77 and a high sensitivity of 0.95 for prediction of antigenic variant of H9N2 subtype (Table 2). For comparison, we also tested the model when it was trained with antigenic data of individual HA subtype. Surprisingly, the model performed slightly better when based on subtypes H1N1 or H5N1, compared to the universal model (Table 2).

Finally, to facilitate public use of the models presented here, a web server was set up by which users can predict antigenic variants for all HA subtypes of influenza A virus (H1-H16). The user can choose between the universal model and the models based on individual subtypes. The web server is publicly available at http://www.computationalbiology.cn/UNIVERSAL/html/antigenicVariantPrediction.php.

## Discussion

This work systematically analyzed and compared the structure and mutation patterns of HA1 protein from nine HA subtypes by protein sequence and structure analysis. A conserved antigenic structure for HA subtypes of influenza A virus was concluded from the observed median correlations between the mutation patterns of HA1 protein, dictated by the highly similar protein structure of HA1 from different HA subtypes. This conclusion is consistent with previous studies, which have shown that different HA subtypes result in immunological cross-reactions^16^,^17^,^18^.

Although there exists a conserved antigenic structure on HA1 protein for influenza A viruses, the detailed forms could be diverse. For example, the sets of ten bands and five bands used here both performed conserved roles in antigenic variation of H1, H3 and H5 influenza viruses based on antigenic analysis and modelling. They are better embodiments of the conserved antigenic structure than the set of randomly separated bands, or the residues. Considering that both sets of bands were defined according to the distances of residues to the top of HA protein, it implicated that the decreased antigenic effects with distance from the top of the HA protein may be conserved and played an important role in modelling the antigenic variant of influenza A viruses.

The ten regional bands defined in this study covered all surface residues on HA1 proteins. Although some of these hardly correlated with antigenic variation, such as E7 and E8 in H3N2 (Table S4), as was reported by Lees *et al*.^6^, many more residues outside the five canonical antigenic epitopes^14^,^15^ on HA1 protein may be related to antigenic variation. Indeed, most of the regional bands defined here were observed to contribute significantly to antigenic variation in influenza H1N1, H3N2 and H5N1 viruses, even when positioned more distant from the top of HA1; this was even true for the most distantly remoted regional band E10, in all three subtypes. Therefore, to capture the antigenic variant at the highest possible level of sensitivity, we used all regional bands to model the antigenic variation of influenza A virus.

For most HA subtypes of influenza A viruses, relatively few antigenic data is available, which hampers the development of specific computational models that can quickly and accurately predict their antigenicity. For this reason, band-based models have to be applied. Although these models performed inferior to those based on residues in the model-training subtypes, they actually performed much better in the testing subtypes, suggesting they can be generally applied with acceptable confidence. A universal model based on regional bands was therefore developed using all the antigenic data of influenza H1, H3 and H5 viruses. When this universal model was used to predict the antigenic relationship between viruses of avian influenza A subtype H9N2, surprisingly, it performed inferior to the models exclusively using the antigenic data of subtypes H1N1 or H5N1 (Table 2). This may be partly explained by the closer genetic relationship of H9 to H1 or H5 as compared to H3. The observation also raises the question how to choose the right model when predicting an antigenic variant for an HA subtype of which few antigenic data are available. On the one hand, a model based on the HA subtype with the closest possible genetic relationship to the target HA subtype could provide a suitable choice; on the other hand, a model built based on more diverse antigenic data would capture more accurately the general mechanisms behind antigenic variation, which are expected be general applicable. For the time being, four different models, including the universal model as well as individual models based on the antigenic data of H1N1, H3N2 and H5N1 subtypes, are available for public use through our web server. Future work will be conducted to fine-tune these models and test their performances with other HA subtypes. In their current state, the publicly available models will nevertheless be helpful for predicting the antigenic variants of multiple HA subtypes of influenza A virus.

## Material and Methods

### Antigenic data

Antigenic data obtained from hemagglutination inhibition (HI) assays were collected from the relevant literature and documents published by the collaborating centers of the World Health Organization (WHO)’s global influenza surveillance network^19^ (details see Supplementary Material). These collected HI data were used for building and testing the computational models to predict the antigenic variants of influenza A viruses. In total, 355 pairs of virus with known HA1 protein sequence and observed antigenic relationship (antigenically similar or distinct) were obtained for subtype H1N1, 791 for H3N2, 293 for H5N1 and 118 pairs were obtained for H9N2. These are all publicly available on the web server, http://www.computationalbiology.cn/UNIVERSAL/html/antigenicVariantPrediction.php.

### HA1 protein sequences

The protein sequences of HA1, the immunogenic part of HA, of different HA subtypes of influenza A viruses were derived from the database of Influenza Virus Resource^20^ as of Dec 17, 2014. Sequences were included only for the HA subtype for which a crystal structure of HA was available in the Protein Data Bank (PDB)^21^ (for details see Supplementary Material) and with more than 100 HA protein sequences available in the database. This resulted in sequences for nine HA subtypes: H1, H2, H3, H5, H6, H7, H9, H10 and H13, which were used in the analysis. After removal of the signal peptide, HA1 protein sequences of each HA subtype were aligned using the software MAFFT 7.127^22^ with manual check. Any laboratory-generated re-assortment sequences, and sequences with a gap ratio greater than 10% in the alignment were removed. The sequences were further pruned by using the software cd-hit^23^ to remove redundant sequences of 100% similarity. The final dataset comprised of 9826 sequences for H1, 425 for H2, 8951 for H3, 3017 for H5, 1010 for H6, 910 for H7, 1826 for H9, 379 for H10 and 110 sequences for H13, which were used in this study. The amino acid positions of these protein sequences are numbered according to that of H3 by structural alignment (for details see Supplementary Information).

The information entropy for each amino acid position on HA1 of different HA subtypes was calculated and normalized by dividing the maximum ln(20). To correct for structural alignment errors, they were further smoothed by the method of moving average with a window size of 11, where the position for calculating information entropy was located at the center with five neighboring residues upstream and five neighboring residues downstream of the central position.

### Defining the regional bands on HA1 protein

The regional bands (or artificial sites) on HA1 protein were defined according to Lees and colleagues^6^. All amino acid residues determined by PISA^24^ to have solvent surface area greater than 10 Å^2^ (Table S6) were allocated to the regional bands depending on the distance of their Cα atoms from the Cα atom of the residue on the top of HA1, i.e., the residue on the extreme-distal end of the HA1, which were identified as 155, 158, 154 and 148 for H1, H3, H5 and H9, respectively (Table S6). Each regional band includes the amino acids covering 10 Ås increments. For example, the first regional band (E1) contains the amino acids at a distance <10 Ås to the top residue of HA1. The amino acids with distance equal to or greater than 100 Ås were all allocated to the tenth regional band (E10).

### Predict the antigenic variant of influenza A virus

The computational model based on the regional bands for predicting antigenic variants of influenza A virus was adapted from work by Du and colleagues^7^. The model is basically a naïve Bayes classifier, which predicts the antigenic relationship (antigenically similar or distinct) between a pair of sequences. To build the computational model, three steps were performed, as previously described^7^: First, the training data set (the antigenic data) was constructed as described above; second, the number of amino acids differences in each regional band between a pair of sequences was extracted as features. These were discretized to avoid overfitting; finally, a naive Bayes classifier was built to predict antigenic relationships. All these steps were achieved with Perl programming.

When building the model for a specific subtype, such as H1N1, the HI dataset of that subtype was used to train the model, while the HI dataset for the other two subtypes (H3N2, H5N1) was used to test the model. For the universal model, the HI dataset of all three subtypes (H1N1, H3N2 and H5N1) were used in combination to train and validate the model. To assess the performance of the universal model, five-fold cross validations were conducted.

For comparison, we also built the computational model based on amino acid residues for predicting antigenic variants from HA sequences, an approach that was adapted from work by Liao and co-workers^25^. It is based on a multiple linear regression model to predict the antigenic distance between sequences. The model makes use of the difference of each non-conserved residue of the HA1 protein between each pair of sequences as features. Considering the different physical-chemical properties of amino acids, the GM4 Scoring method was used here, which was previously reported to perform best^25^. The predicted antigenic distances were discretized into two classes (antigenically similar or distinct) with a cutoff value of 4 (<4 or ≧4).

### Statistical analyses

All the statistical analyses in this work were conducted with the software R version 3.2.1^26^. The package ROCR^27^ was used to plot the receiver operating characteristic (ROC) curve and calculate the area under ROC curve (AUC).

## Additional Information

**How to cite this article:** Peng, Y. *et al*. A universal computational model for predicting antigenic variants of influenza A virus based on conserved antigenic structures. *Sci. Rep.* **7**, 42051; doi: 10.1038/srep42051 (2017).

**Publisher's note:** Springer Nature remains neutral with regard to jurisdictional claims in published maps and institutional affiliations.

## Supplementary Material

Supplementary Information

## Figures and Tables

**Table 1:** Performance of regional band-based and residue-based computational models trained and tested with influenza A virus subtypes H3N2, H1N1 and H5N1.

Subtype (training)	Subtype (testing)	Regional band-based model			Residue-based model		
		Accu	Sen	Spe	Accu	Sen	Spe
H3N2	H3N2*	0.79	0.75	0.84	0.86	0.83	0.89
	H1N1	0.67	0.57	0.78	0.60	0.40	0.81
	H5N1	0.76	0.68	0.89	0.62	0.61	0.64
H1N1	H3N2	0.78	0.78	0.78	0.57	0.41	0.71
	H1N1*	0.74	0.69	0.79	0.83	0.83	0.83
	H5N1	0.75	0.68	0.86	0.73	0.70	0.77
H5N1	H3N2	0.76	0.83	0.68	0.66	0.61	0.70
	H1N1	0.72	0.69	0.76	0.61	0.43	0.80
	H5N1*	0.83	0.85	0.81	0.86	0.88	0.84

^*^Performance in five-fold cross-validations. “Accu”, accuracy; “Sen”, sensitivity; “Spe”, specificity.

**Table 2:** Performance on the antigenic data of avian influenza H9N2 viruses for the regional band-based models built on the antigenic data of H1N1, H3N2, H5N1 and in combination.

Subtypes for training models	Accuracy	Sensitivity	Specificity
Combination of H1N1, H3N2 and H5N1	0.77	0.95	0.26
H1N1	0.80	0.94	0.39
H3N2	0.75	0.93	0.26
H5N1	0.79	0.93	0.39
