# Supplementary material for: A universal computational model for predicting antigenic variants of influenza A virus based on conserved antigenic structures
Source: Sci Rep. 2017 Feb 6;7:42051. doi: 10.1038/srep42051 (PMC5292743; doi:10.1038/srep42051)
Supplement: Supplementary Information [file srep42051-s1.pdf]

Supplementary Information for

A universal computational model for predicting antigenic variants of influenza A virus based on conserved antigenic structures

Yousong Peng[1,*], Dayan Wang[2], Jianhong Wang[3], Kenli Li[3], Zhongyang Tan[1], Yuelong Shu[2], Taijiao Jiang[4, 5,*]

[1] College of Biology, Hunan University, Changsha, 410082, China
[2] National Institute for Viral Disease Control and Prevention, China CDC, Beijing, 102206, China
[3] College of Computer Science and Electronic Engineering, Hunan University, Changsha, 410082, China
[4] Center of System Medicine, Institute of Basic Medical Sciences, Chinese Academy of Medical Sciences & Peking Union Medical College, Beijing, 100005, China
[5] Suzhou Institute of Systems Medicine, Suzhou, Jiangsu, 215123, China

* To whom correspondence should be addressed. YP, Tel: +86-13755111608; Email: pys2013@hnu.edu.cn. TJ, Tel: 86-010-64888427; Email: taijiao@moon.ibp.ac.cn

Supplementary Material and Methods

The HI data

The HI data for human influenza H1N1, H3N2 and avian influenza H5N1 viruses were collected from the relevant literatures and documents published by the collaborating centers of the WHO's global influenza

surveillance network (Table S7). Then, the Archetti-Horsfall distance (dAH), which was reported to be less dependent on antigenic factors than other measures[1], was used to measure the antigenic differences between viral strains. It was calculated as follows:

$$dAH = \sqrt{H_{ii} H_{jj} / H_{ij} H_{ji}},$$

where H_{ij} refers to the HI titer of strain i relative to antisera raised against strain j. When the dAH of a pair of strains was measured in several independent HI assays, the median was taken. When the dAH was equal to or greater than the threshold (defined to be 4 as in Liao's work[2]), the pair of viral strains was considered as antigenic variant pair; otherwise antigenic similar pair. Using the dAH measure, 355, 791, 293 and 118 antigenic pairs were obtained for influenza H1N1, H3N2, H5N1 and H9N2 viruses, respectively.

Structural data and structure alignment

The structural data for 9 HA subtypes were collected from the Protein Data Bank database[3]. 1RUZ, 2WRD, 1HGD, 2IBX, 4WSR, 4N5J, 1JSD, 4XQ5 and 4KPQ were used for H1, H2, H3, H5, H6, H7, H9, H10 and H13, respectively. The alignment between the HA1 structure of these HA subtypes were conducted by the software TM-align[4]. The structures were visualized and manipulated by the software RasMol[5].

Supplementary Tables and Figures

Table S1. The structural similarity measured with TM-score (lower triangular matrix) and RMSD (root-mean-square deviation) (upper triangular matrix) between the HA1 structures of nine HA subtypes. The TM-score ranges between (0, 1], where 1 indicates a perfect match between two structures.

HA subtype	H1	H2	H3	H5	H6	H7	H9	H10	H13
H1	-	1.48	2.23	1.14	1.05	2.41	1.45	2.3	2.02
H2	0.95	-	2.39	1.55	1.6	2.43	2.03	2.19	2.16
H3	0.88	0.87	-	2.02	2.19	1.7	2.03	2	2.39
H5	0.97	0.95	0.89	-	1.24	2.18	1.62	2.08	2.16
H6	0.97	0.94	0.88	0.96	-	2.42	1.36	2.27	2.09
H7	0.87	0.87	0.92	0.89	0.87	-	2.28	1.17	2.65
H9	0.95	0.91	0.89	0.94	0.95	0.88	-	2.2	1.91
H10	0.88	0.89	0.9	0.9	0.89	0.96	0.88	-	2.67
H13	0.92	0.9	0.87	0.91	0.91	0.86	0.92	0.85	-

Table S2. Correlation (Pearson Correlation Coefficient) between the information entropy of amino acid positions on HA1 protein between nine HA subtypes in terms of moving average position information entropy.

HA subtype	H1	H2	H3	H5	H6	H7	H9	H10	H13
H1	1.00	0.32	0.56	0.56	0.62	0.33	0.49	0.12	0.59
H2	0.32	1.00	0.35	0.64	0.20	0.04	0.26	-0.05	0.48
H3	0.56	0.35	1.00	0.58	0.58	0.22	0.56	0.12	0.59
H5	0.56	0.64	0.58	1.00	0.60	0.28	0.49	0.10	0.50
H6	0.62	0.20	0.58	0.60	1.00	0.34	0.44	0.11	0.37
H7	0.33	0.04	0.22	0.28	0.34	1.00	0.28	0.69	0.05
H9	0.49	0.26	0.56	0.49	0.44	0.28	1.00	0.00	0.58
H10	0.12	-0.05	0.12	0.10	0.11	0.69	0.00	1.00	-0.04
H13	0.59	0.48	0.59	0.50	0.37	0.05	0.58	-0.04	1.00

Table S3. The amino acid positions significantly associated with antigenic variation in human influenza H3N2, H1N1 and avian influenza H5N1 viruses. The table shows the Spearman Correlation Coefficient (SCC) between the changes of amino acid residues and the antigenic variation, and its related p-value. The amino acid positions of HA1 protein sequences for each subtype are numbered according to that of H3 by structural alignment. [a], the canonical five antigenic epitopes in H3N2 viruses[6,7]; [b], other region besides for the epitopes; [c], p-value greater than 0.05, or not applied.

Pos	Ep[a]	H3N2		H1N1		H5N1	
		SCC	p-value	SCC	p-value	SCC	p-value
1	O[b]	-0.09	1.42e-02	-[c]	-	-	-
2	O	0.31	0	-	-	-	-
3	O	0.19	5.72e-08	-	-	-	-
7	O	0.08	1.75e-02	-	-	-	-
9	O	0.23	7.70e-11	-	-	-	-
10	O	0.16	6.47e-06	-	-	-	-
20	O	-0.09	1.13e-02	-	-	-	-
21	O	0.14	9.21e-05	-	-	-	-
25	O	0.22	1.09e-09	-	-	-	-
29	O	-0.09	1.44e-02	-	-	-	-
31	O	0.25	2.45e-12	-	-	-	-
33	O	-0.13	2.67e-04	-	-	-	-
34	O	0.16	3.55e-06	-	-	-	-
45	C	-0.13	2.57e-04	-	-	-	-
47	C	0.15	3.29e-05	-	-	-	-
48	C	-0.10	4.77e-03	-	-	-	-
50	C	0.33	0	-	-	0.15	9.32e-03
53	C	0.20	9.05e-09	0.30	1.19e-08	-	-
54	C	0.27	1.16e-14	-	-	-	-

56	O	-	-	0.13	1.62e-02	-	-
62	E	0.32	0	-	-	-	-
63	E	0.26	5.55e-14	0.26	7.51e-07	-	-
65	O	-	-	0.22	3.70e-05	-	-
67	E	-0.11	2.37e-03	-	-	-	-
75	E	0.22	4.10e-10	-	-	-	-
78	E	0.17	1.18e-06	-	-	-	-
79	O	-0.09	1.42e-02	-	-	-	-
80	E	0.11	1.85e-03	0.33	3.11e-10	-	-
82	E	0.24	3.34e-12	0.14	1.08e-02	-	-
83	E	0.40	0	-	-	-	-
88	E	-	-	0.28	1.43e-07	-	-
93	O	-	-	0.11	4.86e-02	-	-
94	E	0.10	3.62e-03	-	-	-	-
96	D	-	-	0.15	4.20e-03	0.21	4.04e-04
101	O	-	-	0.16	2.02e-03	-	-
103	D	-	-	-0.12	2.83e-02	-	-
104	O	0.08	1.75e-02	-	-	-	-
112	O	-0.15	2.42e-05	-	-	-	-
121	D	0.19	6.31e-08	-	-	0.27	2.19e-06
122	A	0.13	2.57e-04	-	-	0.16	7.28e-03
123	O	-	-	-	-	0.16	7.28e-03
124	A	0.19	7.19e-08	-	-	-	-
126	A	0.20	2.20e-08	-	-	-	-
129	B	0.16	6.47e-06	0.36	1.40e-12	-	-
130	A	-	-	-	-	0.16	7.28e-03
131	A	0.31	0	0.17	1.12e-03	-	-
132	A	0.16	6.47e-06	-	-	0.22	1.35e-04
133	A	0.35	0	0.18	7.32e-04	-	-
135	A	0.29	2.22e-16	-	-	-	-
136	O	-	-	0.17	1.14e-03	-	-
137	A	0.35	0	-	-	0.32	3.27e-08
138	A	-0.07	4.82e-02	0.19	2.51e-04	-	-
139	O	0.11	1.85e-03	-	-	-	-
140	A	-	-	0.14	8.30e-03	-	-
141	O	-	-	0.14	8.309e-03	-	-
142	A	-	-	0.23	1.60e-05	-	-
143	A	0.29	0	0.14	8.30e-03	-	-

144	A	0.24	4.05e-12	0.35	7.80e-12	-	-
145	A	0.30	0	-	-	-	-
146	A	0.30	0	-	-	-	-
148	O	0.12	7.24e-04	-	-	0.62	0
152	A	-	-	-	-	0.27	2.19e-06
155	B	0.42	0	-	-	-	-
156	B	0.35	0	0.14	7.93e-03	-	-
157	B	0.27	8.44e-15	-	-	-	-
158	B	0.42	0	0.13	2.90e-03	-	-
159	B	0.25	8.61e-13	-	-	-0.12	3.59e-02
160	B	0.27	4.22e-15	0.11	4.86e-02	-	-
163	B	0.24	3.34e-12	-	-	0.28	1.76e-06
164	B	0.26	7.64e-14	-	-	0.49	0
165	B	-0.07	4.67e-02	0.14	8.30e-03	-	-
166	O	-	-	0.11	4.08e-02	-	-
169	O	-0.09	1.42e-02	-	-	-	-
171	D	-	-	0.11	3.87e-02	-	-
172	D	0.25	1.42e-12	-	-	-	-
173	D	0.19	4.76e-08	-	-	0.32	1.36e-08
174	D	0.277	1.78e-15	-	-	-	-
182	D	0.10	6.52e-03	-	-	0.28	1.58e-06
183	O	0.08	3.59e-02	-	-	-	-
186	B	-	-	0.22	4.37e-05	-	-
189	B	0.45	0	0.23	1.52e-05	0.33	8.99e-09
190	B	0.16	1.03e-05	0.17	1.46e-03	-	-
192	B	-	-	0.16	3.14e-03	0.34	2.16e-09
193	B	0.23	1.16e-10	-	-	0.25	1.86e-05
194	B	-	-	0.24	3.54e-06	-	-
196	B	-	-	0.24	3.79e-06	-	-
197	B	0.32	0	-	-	-	-
198	B	-	-	-	-	0.26	5.21e-06
200	O	-	-	-	-	0.20	4.81e-04
201	D	0.23	1.21e-10	-	-	-	-
202	O	0.18	2.71e-07	-	-	-	-
207	D	0.22	3.01e-10	-	-	-	-
208	D	0.14	1.26e-04	0.20	1.65e-04	0.25	1.81e-05
209	D	0.18	6.40e-07	-	-	-	-
210	O	-	-	0.14	1.08e-02	-	-

213	D	0.28	2.66e-15	-	-	-	-
214	D	0.10	5.11e-03	-	-	-	-
216	D	0.12	8.63e-04	-	-	-	-
217	D	0.28	2.44e-15	-	-	-	-
219	D	-	-	0.20	1.16e-04	-	-
222	O	0.16	5.42e-06	-	-	-	-
223	O	-0.09	1.0e-02	-	-	-	-
225	O	0.09	1.49e-02	0.21	8.61e-05	0.16	6.44e-03
226	D	0.08	1.95e-02	-	-	-	-
227	D	-0.07	4.05e-02	0.23	1.60e-05	-	-
229	D	0.09	8.54e-03	-	-	-	-
230	D	0.11	1.47e-03	-	-	-	-
233	O	0.08	2.04e-02	-	-	-	-
240	D	0.11	1.85e-03	-	-	-	-
242	D	0.19	1.19e-07	-	-	-	-
244	D	0.28	2.67e-15	-	-	-	-
246	D	-0.11	2.60e-03	-	-	0.26	5.21e-06
247	D	0.07	4.84e-02	-	-	-	-
248	D	0.20	7.70e-09	-	-	-	-
260	E	0.26	1.53e-13	-	-	-	-
261	E	-	-	0.18	7.01e-04	-	-
262	E	0.24	5.81e-12	-	-	-	-
264	O	-	-	0.15	4.20e-03	-	-
269	O	-0.11	2.94e-03	-	-	-	-
272	O	-	-	-	-	0.20	7.58e-04
273	C	-	-	0.29	2.56e-08	-0.21	2.51e-04
274	O	-	-	0.12	2.57e-02	-	-
275	C	0.18	6.12e-07	-	-	-	-
276	C	0.26	5.68e-14	-	-	0.31	5.73e-08
278	C	0.24	1.06e-11	-	-	-	-
279	C	-	-	0.17	1.17e-03	-	-
280	C	-0.11	2.44e-03	-	-	-	-
285	O	-	-	-0.12	2.83e-02	-	-
297	C	-	-	0.22	3.70e-05	-	-
299	C	0.21	2.21e-09	-	-	-	-
300	C	-	-	0.14	8.30e-03	-	-
307	C	0.24	1.42e-11	-	-	-	-
308	C	0.13	4.29e-04	-	-	-	-

310	C	-0.09	1.44e-02	-	-	-	-
312	C	-0.13	2.27e-04	0.16	2.54e-03	-	-
326	O	-0.10	6.76e-03	-	-	-	-
328	O	-	-	0.11	3.81e-02	-	-

Table S4. The correlation (Pearson Correlation Coefficient (PCC)) between the antigenic variation and the changes of ten regional bands (E1~E10) in influenza H1N1, H3N2 and H5N1 viruses. "**", p-value < 0.01; "*", p-value < 0.05.

Regional bands	H3N2	H1N1	H5N1
E1	0.67**	0.54**	0.54**
E2	0.64**	0.51**	0.63**
E3	0.53**	0.46**	0.58**
E4	0.57**	0.43**	0.48**
E5	0.53**	0.13*	0.53**
E6	0.56**	0.44**	0.35**
E7	0.06	0.24**	0.20**
E8	0.00	0.18**	0.22**
E9	0.33**	-0.07	0.15*
E10	0.42**	0.21**	0.12*

Table S5. The predictive accuracies of the band-based and residue-based computational models trained and tested in influenza H3N2, H1N1 and H5N1 viruses. *, performance in five-fold cross-validations. “Original model” refers to the original model based on ten regional bands. “Alternative model 1” refers to the model using another partitioning of the HA1 protein, the model (M4) based on which achieved best performance in Lees’s work[8]. “Alternative model 2” refers to the model for which the surface residues on HA1 protein were randomly separated into five bands.

To evaluate the robustness of these models, for the “Alternative model 2”, the surface residues on HA1 protein were randomly separated into five bands for 100 times; while for the other models, the training data were randomly sampled with a bootstrap method, which were also repeated 100 times. For each time, a model was built and tested. The average and the standard deviation (in brackets) for the predictive accuracies in these models were calculated and listed in the table.

Subtype (training)	Subtype (testing)	Band-based models			Residue-based model
		Original model	Alternative model 1	Alternative model 2	
H3N2	H3N2*	0.81(0.01)	0.81(0.02)	0.79(0.01)	0.88(0.01)
	H1N1	0.67(0.01)	0.67(0.02)	0.64(0.04)	0.61(0.04)

	H5N1	0.80(0.02)	0.80(0.02)	0.73(0.07)	0.60(0.07)
H1N1	H1N1*	0.74(0.03)	0.74(0.03)	0.72(0.02)	0.86(0.02)
	H3N2	0.72(0.02)	0.75(0.02)	0.71(0.03)	0.61(0.04)
	H5N1	0.82(0.03)	0.82(0.03)	0.76(0.05)	0.70(0.04)
H5N1	H5N1*	0.84(0.02)	0.84(0.02)	0.84(0.01)	0.88(0.02)
	H3N2	0.75(0.01)	0.75(0.01)	0.71(0.03)	0.68(0.05)
	H1N1	0.70(0.01)	0.70(0.02)	0.65(0.04)	0.58(0.04)

Table S6. The solvent surface area (SSA) for each residue on the HA1 protein of influenza H1, H3, H5 and H9 viruses, and the distance of the residue's Cα atoms from the Cα atom of the residue on the top of HA1 protein. The amino acid position numbering of HA1 protein sequences for each subtype are aligned to that of H3 by structural alignment.

H3N2			H1N1			H5N1			H9N2		
Res. Num	SSA	Dist	Res. Num	SSA	Dist	Res. Num	SSA	Dist	Res. Num	SSA	Dist
1	192.54	130.4	-	-	-	-	-	-	-	-	-
2	103.28	131.47	-	-	-	-	-	-	-	-	-
3	150.13	134.9	-	-	-	-	-	-	-	-	-
4	102.36	135.1	-	-	-	-	-	-	-	-	-
5	64.52	131.5	-	-	-	-	-	-	-	-	-
6	137.25	132.4	-	-	-	-	-	-	-	-	-
7	76.16	129.62	-	-	-	-	-	-	-	-	-
8	97.35	128.56	-	-	-	-	-	-	-	-	-
9	36.49	125.35	-	-	-	-	-	-	-	-	-

10	53.17	121.91	-	-	-	-	-	-	-	-	-
11	3.39	118.3	1	199.59	119.49	1	50.03	118.9	1	202.49	120.49
12	36.29	115.19	2	134.12	116.31	2	53.83	115.54	2	178.6	117.23
13	0.01	111.73	3	153.13	112.75	3	0	112.49	3	160.38	113.73
14	21.91	108.75	4	118.77	109.68	4	6.55	109.39	4	96.04	110.77
15	3.98	105.29	5	160.11	106.4	5	0.67	106.18	5	163.98	107.31
16	0.49	102.37	6	66.77	103.31	6	0	103.18	6	64.48	104.46
17	0.01	98.66	7	144.75	99.58	7	0.1	99.51	7	147.43	100.76
18	18.85	96.71	8	102.46	97.28	8	18.63	97.01	8	101.09	98.52
19	4.45	95.21	9	36.59	95.89	9	1.93	95.96	9	55.05	97.58
20	31.11	92.99	10	43.57	94.17	10	39.07	93.96	10	47.26	95.22
21	83.55	93.28	11	135.57	92.34	11	146.12	92.03	11	151.94	93.67
22	111.91	89.57	12	25.61	88.97	12	28.07	88.77	12	33.08	90.33
23	23.64	88.12	13	117.11	87.23	13	116.49	87.42	13	125.5	88.61
24	42.91	84.89	14	57.69	84.44	14	71.79	84.72	14	62.65	86.32
25	79.86	84.42	15	33.43	84.41	15	69.19	84.42	15	52.65	86.35
26	5.35	82.63	16	6.89	83.12	16	0.11	83.23	16	9.96	84.79
27	62.73	81.94	17	79.46	83.12	17	35.41	82.93	17	97.17	84.56
28	0.47	83.87	18	71	84.79	18	8.67	84.67	18	76.81	85.82
29	13.98	84.16	19	153.12	84.89	-	-	-	19	186.67	85.87
30	6.43	87.13	20	170.93	88.42	19	3.28	84.81	20	133.69	89.48
31	55.28	86.62	21	81.56	88.11	20	27.93	88.18	21	70.64	89.31
32	82.59	83.56	22	159.7	85.52	21	75.92	87.94	22	91.64	86.86
33	151.97	85.13	23	109.53	85.55	22	130.6	85.16	23	101.71	87.06
-	-	-	-	-		23	92.23	85.23	-	-	-
34	25.2	87.67	24	13.07	88.06	24	3.37	87.93	24	9.71	89.58
35	36.54	88.4	25	42.6	88.64	25	40.79	88.64	25	57.19	90.6
36	0	88.09	26	6.68	88.79	26	0	88.61	26	4.85	90.2
37	17.24	88.83	27	7.23	89.85	27	2.83	89.82	27	5.89	91.16
38	77.52	85.23	28	94.31	86.7	28	71.16	86.56	28	90.26	87.84
39	20.77	81.76	29	23.7	83.23	29	24.06	83.18	29	28.02	84.4
40	43.87	78.23	30	68.33	79.62	30	68.31	79.86	30	97.44	80.84
41	52.5	74.97	31	55.95	76.38	31	50.54	76.76	31	54.83	77.69
42	1.33	72.52	32	37.3	73.79	32	1.75	74.13	32	30.79	75.2
43	2.29	69.58	33	8.74	71.07	33	0.23	71.15	33	4.91	72.33
44	25.85	67.27	34	27.73	68.64	34	21.71	68.62	34	33.46	69.96
45	63.44	66.36	35	92.31	67.24	35	100.29	67.66	35	69.68	68.73
46	59.89	64.85	36	69.61	66.29	36	92.34	66.11	36	70.95	67.14
47	35.75	61.81	37	87.37	63.11	37	51.34	62.66	37	62.31	63.6

48	89.09	61.74	38	54.41	61.28	38	62.17	61.14	38	58.59	62.03
49	32.58	58.73	39	27.34		39	30.47	58.35	39	24.28	59.59
50	82.54	56.59	40	63.13	58.96	40	93.88	55.75	40	62.8	56.64
51	0	54.44	41	1.15	56.31	41	0	52.89	41	1.23	53.69
52	6.06	55.96	42	0	53.4	42	0	53.9	42	1.16	54.51
53	56.85	53.38	43	52.46	54.59	43	22.01	53.01	43	3.28	53.65
54	73.85	55.48	44	20.73	53.82	44	24.82	54.18	44	5.28	55.38
-	-	-	45	178.53	54.61	45	94.34	56.34	45	139.41	56.39
55	61.24	55.8	46	48.2	54.84	46	51.81	54.61	46	90.46	52.76
56	13.98	52.46	47	79.33	51.12	47	47.7	51.12	47	25.62	51.49
57	117.73	49.19	48	39.47	49.85	48	94.55	49.02	48	117.38	49.74
58	24.76	48.18	49	17.66	47.67	49	2.54	47.02	49	21.34	47.69
59	16.54	44.57	50	13.9	44	50	10.41	43.32	50	6.86	43.99
60	42.16	44.43	51	89.47	43.54	51	12.02	43.79	51	53.38	44.17
61	0	41.11	52	0.16	40.27	52	0	40.4	52	3.18	41.12
62	105.15	41.79	53	18.35	41.69	53	148.87	41.63	53	75.7	41.73
63	48.24	39.18	54	84.91	39.14	54	61.99	39.23	54	71.87	38.32
64	1.14	36.87	55	0.17	36.81	55	5.25	36.66	55	3.53	36.23
65	29.98	37.34	56	28.07	36.99	56	1.6	36.66	56	21.59	36.97
66	0.02	36.92	57	0	36.25	57	0	36.44	57	0	36.9
67	0.06	33.69	58	6.02	32.7	58	0.51	32.98	58	21.46	33.69
68	0.63	31.98	59	0	31.96	59	0	31.9	59	0	32.12
69	5.6	33.39	60	16.89	33.62	60	3.28	33.17	60	8.1	33.01
70	0.64	31.89	61	0.29	31.91	61	0.54	31.75	61	0	31.89
71	1.17	28.38	62	0.17	28.41	62	0	28.16	62	0	28.38
72	2.74	28.35	63	2	28.74	63	8.66	28.66	63	3.35	28.17
73	0.57	28.56	64	7.15	28.71	64	1.57	28.52	64	3.91	28.2
74	19.2	28.47	65	2.55	28.86	65	1.36	27.84	65	13.7	27.01
75	48.04	32.06	66	104.71	32.46	66	106.32	31.3	66	56.11	30.56
76	1.16	33.22	67	5.26	33.7	67	4.58	32.87	67	5.92	32.2
77	44.74	31.91	68	47.08	33.41	68	45.08	32.55	68	60.47	31.17
78	125.54	35.6	69	134.2	37.17	69	116.09	36.3	69	159.85	34.87
79	12.38	36.03	70	14.26	36.93	70	9.41	35.57	70	20.43	34.89
80	106.28	33.71	71	67.23	34.46	71	84.08	32.75	71	53.8	32.07
-	-	-	-	-	-	-	-	-	72	78.34	34.67
81	81.35	35.64	72	124.76	37.48	72	93.52	35.21	73	21.62	36.64
82	47.79	38.67	73	31.73	39.53	73	20.85	38.67	74	103.15	40.09
-	-	-	74	58.01	40.32	74	102.98	42.03	-	-	-
83	38.87	41.49	75	65.62	43.66	75	75.61	44.06	75	139.56	43.24

84	7.16	44.34	76	6.55	45.67	76	2.05	45.6	76	7.63	45.72
85	27.59	47.42	77	16.94	48.67	77	7.85	48.8	77	0	48.95
86	0.6	47.01	78	0	47.18	78	0	47.56	78	0.5	47.79
87	0	43.95	79	1.34	44.2	79	0.16	44.61	79	0	45.13
88	0	45.34	80	1.17	44.94	80	3.11	45.34	80	0	45.83
89	14.61	43	81	18.69	43.18	81	4.42	43.2	81	15.92	43.48
90	33.86	44.07	82	29.18	44.68	82	58.93	43.89	82	35.76	44.58
91	81.32	44.25	83	81.05	45.09	83	54.06	44.52	83	97.34	44.54
92	156	42.41	84	93.01	42.02	84	129.2	41.57	84	62.33	42.34
93	22.49	39.2	85	20.74	39.61	85	26.99	39.16	85	12.41	39.58
94	92.07	36.14	86	127.05	36.8	86	121.91	36.9	86	115.94	37.05
95	45.02	32.46	87	37.06	33.96	87	31.09	34.12	87	42.99	33.71
96	65.47	30.15	88	3.39	31.84	88	48.6	33.13	88	10.98	32.61
-	-	-	89	50.21	28.83	89	27.97	29.67	89	34.85	29.56
97	2.35	26.47	90	8.86	27.49	90	13.19	28.21	90	17.37	27.99
98	4.47	23.92	91	6.68	24.45	91	6.82	25.47	91	14.04	25.35
99	7.11	26.25	92	11.03	26.07	92	7.89	27.76	92	2.66	27.41
100	42.75	26.76	93	27.34	26.15	93	26.13	28.13	93	19.97	28.27
101	27.88	29.49	94	89.03	28.43	94	67.65	30.27	94	125.34	30.98
102	14.74	30.87	95	29.65	29.81	95	18.6	31.12	95	14.67	31.58
103	78.82	32.32	96	42.84	30.86	96	60.31	32.19	96	62.42	33
104	69.97	35.26	97	84.1	34.33	97	61.79	35.66	97	92.05	36.44
105	83.9	35.42	98	43.49	34.7	98	31.76	35.92	98	48.83	36.48
106	41.36	39.02	99	125.27	38.52	99	27.22	39.66	99	131.03	40.21
107	1.72	38.63	100	104.18	38.69	100	6.11	39.98	100	110.22	39.93
108	0.01	35.75	101	0.17	36.67	101	5.63	37.85	101	0	37.07
109	12.03	37.83	102	59.77	39.25	102	16.97	40.02	102	30.83	39.13
110	13.2	40.86	103	124.73	42.03	103	19.63	42.9	103	55.98	42.25
111	6.69	39.31	104	56.13	40.57	104	45.75	41.77	104	71.04	40.73
112	2.33	37.89	105	2.73	40.43	105	2.92	40.9	105	0	39.39
113	0	41.08	106	32.59	44.07	106	23.9	44.56	106	3.32	42.68
114	27.54	43.36	107	42.8	45.1	107	144.54	45.72	107	53.52	44.73
115	6.64	41.22	108	0.24	42.94	108	7.98	43.57	108	7.78	43.24
116	4.98	41.92	109	36.03	44.87	109	10.65	44.51	109	27.26	44.47
117	2.98	39.07	110	22.3	42.35	110	39.92	41.76	110	27.5	41.29
118	1.38	35.58	111	6.98	39.07	111	10.13	38.06	111	7.06	37.54
119	40.28	35.2	112	78.79	37.01	112	95.13	36.77	112	96.79	35.32
120	18.17	31.57	113	72.16	33.55	113	24.22	32.97	113	52.57	31.52
121	72.4	30.4	114	35.59	31.18	114	46.83	30.73	114	23.76	29.84

122	100.55	26.8	115	62.55	27.53	115	82.88	27.05	115	76.22	26.21
123	31.7	25.59	116	12.62	26.7	116	16.19	25.87	116	4.73	25.08
124	69.52	23.05	117	3.59	23.08	117	3.85	22.08	117	2.56	21.41
125	19.51	20.53	118	44.86	22.91	118	47.3	20.89	118	54.4	20.18
-	-	-	119	52.95	19.98	119	100.96	18.18	119	29.58	16.79
-	-	-	120	115.2	19.7	120	104.93	17.54	-	-	-
-	-	-	121	84.36	20.43	-	-	-	-	-	-
126	95.94	17.68	122	12.14	19.41	121	42.23	17.29	120	125.38	16.14
127	13.65	14.48	123	12.56	15.69	122	17.61	13.85	121	85.21	16.44
128	100.19	12.45	124	81.27	14.21	123	101.41	12.3	122	18.67	13.16
129	41.37	8.9	125	91.21	10.94	124	52.05	9.26	123	108.73	10.05
130	14.54	8.37	126	10.3	10.11	125	13.05	8.67	124	3.43	8.25
131	64.5	8.62	127	32.41	10.41	126	60.27	8.99	125	31.67	8.82
132	84.63	12.44	128	24.76	13.88	127	13.41	12.57	126	76.17	12.61
133	86.96	15.12	129	83.76	13.94	128	71.95	13.99	127	70.97	13.95
-	-	-	130	157.84	14.94	129	115.72	12.57	-	-	-
134	19.8	14.1	131	10.1	17.99	130	5.43	14.81	128	19.99	14.33
135	136.95	17.24	132	79.3	19.82	131	74.62	17.79	129	78.81	17.7
136	12.92	19.13	133	17.54	23.13	132	10.11	20.24	130	13.15	20.27
137	106.21	21.78	134	71.32	24.62	133	83.42	23.06	131	93.08	23.12
138	11.43	24.13	135	5.85	25.05	134	17.61	25.24	132	23.37	25.34
139	0	24.33	136	2.85	25.95	135	0.2	25.09	133	0.65	24.92
140	89.85	25.21	137	51.48	28	136	57.55	26.17	134	71.13	25.92
141	75.66	25.57	138	109.58	30.48	137	75.67	27.13	-	-	-
142	60.25	27.67	139	107.86	29.03	138	148.79	29.08	-	-	-
143	134.25	26.68	140	72.95	25.35	139	76.31	27.96	-	-	-
144	17.17	22.95	141	68.54	22.68	140	164.79	24.16	-	-	-
145	22.32	21.18	142	48.9	22.29	141	48.67	22	135	107.36	22.45
146	4.54	21.34	143	18.29	22.12	142	15.4	21.75	136	26.51	21.46
147	11.69	21.5	144	13.73	24.27	143	12.57	22.1	137	7.96	21.41
148	2.19	24.03	145	5.14	25.47	144	2.56	24.24	138	1.72	23.59
149	29.96	24.7	146	56.68	25.46	145	68.07	24.49	139	62.57	24.34
150	28.53	25	147	3.84	22.19	146	4.69	25.02	140	0.74	24.87
151	0.72	21.86	148	2.83	18.66	147	4.5	22.09	141	0.24	21.52
152	3.43	18.1	149	8.62	15.73	148	7.16	18.36	142	44.9	17.78
153	22.18	15.62	150	20.2	12.62	149	20	16.13	143	21.56	15.88
154	0	12.52	151	0	9.23	150	0	12.87	144	0	12.9
155	19.95	9.51	152	12.31	5.62	151	18.14	9.83	145	15.57	10.03
156	63	6.11	153	41.91	5.86	152	51.57	6.49	146	43.69	6.63

157	53.52	3.76	154	106.33	3.81	153	92.12	3.85	147	108.45	3.81
158	66.26	0	155	62.93	0	154	148.72	0	148	129.08	0
159	88.42	3.76	156	73.3	3.81	155	63.81	3.84	149	127.07	3.81
160	81.39	5.95	157	59.02	6.35	156	75.65	5.57	150	41.37	5.41
161	2.08	8.45	158	10.58	9.48	157	5.58	8.51	151	3.1	8.34
162	44.16	11.11	159	52.51	12.82	158	52.9	10.69	152	67.14	10.77
163	88.06	14.74	160	125.05	14.56	159	71.24	14.25	153	95.93	14.28
164	6.22	15.63	161	11.38	18.34	160	4.81	15.43	154	5.09	14.91
165	86.9	19.2	162	72.08	20.49	161	148.62	18.79	155	58.16	18.43
166	21.56	20.65	163	66.96	24.25	162	63.76	20.3	156	3.66	20.04
167	76.66	24.34	164	54.9	27.27	163	69.25	23.98	157	102.07	23.76
168	29.05	26.59	165	32.86	30.99	164	36.21	26.73	158	39.05	26.23
169	62.07	30.32	166	68.86	33.61	165	77.71	30.39	159	57.26	29.96
170	0.38	33.06	167	3.32	35.99	166	0.57	32.96	160	0.49	32.46
171	130.34	35.23	168	121	37.75	167	120.44	35.14	161	126.65	34.75
172	57.93	37.2	169	86.88	40.77	168	25.16	36.7	162	104.1	36.01
173	131.18	40.27	170	68.6	39.91	169	148.69	39.89	163	57.73	39.19
174	52.15	39.21	171	65.19	37.6	170	49.14	39.36	164	98.44	38.62
175	37.81	37.14	172	45.38	34.01	171	27.15	37.87	165	42.08	36.78
176	3.33	33.64	173	0	32.53	172	0	34.29	166	0	33.44
177	4.05	32.34	174	0.66	28.8	173	3.07	33.08	167	2	32.7
178	0.28	28.56	175	0.16	26.96	174	0	29.46	168	0.31	28.99
179	6.08	27.35	176	3.09	23.44	175	0.14	27.46	169	0	26.99
180	0.26	24	177	0	21.71	176	0.59	24.28	170	0.62	24.04
181	0	22.87	178	0	19.63	177	0	23	171	0	23.13
182	0	20.87	179	0.5	17.88	178	1.06	21.26	172	2.84	21.78
183	0.27	19.32	180	6.78	18.58	179	2.36	19.96	173	2.05	20.13
184	4.72	20.97	181	17.87	16.82	180	3.86	21.56	174	14.09	21.59
185	0	19.09	182	0.5	19.24	181	0	19.86	175	1.34	19.9
186	16.07	21.44	183	34.74	17.47	182	33.19	22.34	176	30.89	22.47
187	63.82	20.03	184	67.72	15.17	183	70.89	21.03	177	59.73	20.99
188	94.75	18.33	185	45.61	13.33	184	84.4	19.55	178	87.48	19.15
189	163.25	16.09	186	103.37	12.51	185	63.63	16.65	179	95.54	16.76
190	38.8	15.24	187	50.66	11.39	186	44.06	15.99	180	51.8	15.75
191	0.39	14.72	188	1.76	8.44	187	1.89	15.14	181	0	15.54
192	53.17	12.15	189	86.43	7.83	188	56.65	12.55	182	57.5	13.14
193	52.4	10.45	190	65.88	8.46	189	142.55	11.38	183	104.47	11.31
194	42.34	10.11	191	41.59	7.47	190	29.25	11	184	40.68	10.94
195	0	10.46	192	0.16	5.69	191	0	10.18	185	0	11.04

196	47	9.73	193	62.46	8.64	192	61.76	9.65	186	64.52	10.26
197	54.38	13.1	194	67.13	11.54	193	32.62	12.56	187	70.4	13.93
198	70.06	16.01	195	64.68	14.14	194	104.31	15.93	188	62.19	17.32
199	83.52	18.08	196	138.66	13.59	195	119.83	18.6	189	102.09	19.16
200	3.58	17.23	197	8.47	16.61	196	8.87	18.07	190	0.7	18.31
201	37.72	19.74	198	37.21	18.32	197	53.12	20.31	191	66.08	20.54
202	0	20.8	199	0.67	21.45	198	0	20.98	192	1.82	20.79
203	0.22	23.67	200	29.87	23.93	199	2.64	23.71	193	18.67	23.61
204	0.33	25.29	201	2.64	27.09	200	0	25.9	194	0	25.26
205	0.41	28.36	202	18.73	30.17	201	0.26	28.69	195	32.32	28
206	3.35	31.1	203	31.32	33.79	202	7.28	31.67	196	24.91	30.65
207	115.63	33.96	204	93.74	34.83	203	30	35.17	197	112.02	33.99
208	150.48	35.78	205	123.16	31.5	204	118.34	36.02	198	100.85	35.4
209	50.12	33.47	206	38.19	29.36	205	36.08	32.66	199	44.26	32.81
210	28.34	30.24	207	137.72	27.11	206	21.32	31.46	200	122.07	30.73
211	65.73	28.59	208	110.75	23.88	207	29.16	28.88	201	71.05	28.82
212	29.59	26.09	209	168.97	21.88	208	76.66	26.18	202	85.11	26.3
213	26.15	25.05	210	17.69	19.85	209	22.31	24.71	203	21.08	24.68
214	72.91	23.03	211	88.24	18.36	210	86.82	23.05	204	112.44	23.38
215	16.55	21.82	212	27.54	21.03	211	21.81	21.77	205	18.93	22.02
216	30.27	23.71	213	125.62	19.49	212	90.84	24.14	206	95.04	24.11
217	20.95	22.36	214	59.53	22.35	213	39.36	22.94	207	43.24	22.67
218	2	24.87	215	50.78	24.39	214	2.75	26.04	208	20.63	25.2
219	25.04	26.16	216	100.42	26.41	215	67.39	27.53	209	96	26.58
220	4.56	28.11	217	55.58	28.57	216	8.19	29.35	210	60.19	29.01
221	5.89	30.24	218	108.81	27.44	217	17.25	31.03	211	106.18	31.64
222	173.75	28.74	219	144.31	28.32	218	151.27	29.31	212	112.07	30.56
223	25.75	28.87	220	65.7	28.02	219	54.08	30.22	213	59.47	30.43
224	94.34	27.74	221	152.79	25.97	220	93.62	29.29	214	90.1	29.5
225	36.27	25.72	222	106.94	23.54	221	54.55	27.52	215	61.16	28.27
226	40.06	23.97	223	36.76	23.35	222	39.28	25.31	216	45.1	25.73
227	24.78	24.72	224	25.45	20.92	223	18.07	25.25	217	78.09	25.92
228	2.23	22.31	225	1.99	22.3	224	2.99	23.17	218	2.06	23.37
229	8.86	23.75	226	44.45	21.98	225	8.26	24.46	219	35.94	24.41
230	0.45	22.93	227	0	24.34	226	0.28	23.73	220	0	23.73
231	2.58	25.73	228	30.58	25.66	227	28.9	26.11	221	48.86	26.33
232	1.4	26.87	229	0.83	27.19	228	0	26.95	222	0.62	27.18
233	33.07	28.15	230	11.16	29.48	229	19.98	28.51	223	27.07	28.63
234	49.63	29.81	231	41.7	31.66	230	30.79	30.39	224	44.09	30.21

235	6.66	30.63	232	13.4	34.24	231	20.61	32.4	225	20.96	32.14
236	34.17	33.45	233	19.73	33.49	232	42.97	34.86	226	51.35	33.91
237	0.8	33.33	234	2.51	36.8	233	0.71	33.86	227	0.17	33.1
238	42.21	36.8	235	83.16	38.17	234	110.35	37.16	228	114.03	36.51
239	56.15	37.84	236	80.78	36.89	235	45.77	37.5	229	66.49	37.31
240	53.18	37.11	237	47.82	34.42	236	132.27	36.8	230	53.31	36.28
241	4.32	34.59	238	25.5	30.69	237	21.31	34.45	231	21.45	33.98
242	18.09	30.85	239	45.74	27.89	238	19.47	30.76	232	31.65	30.36
243	0.75	27.84	240	0.6	24.58	239	0.07	28.24	233	0	27.61
244	14	25.09	241	46.66	21.28	240	23.39	25.55	234	100.94	24.8
245	0.72	22.07	242	0.67	18.72	241	3.99	22.34	235	0.64	21.87
246	4.53	20.2	243	54.28	15.72	242	38.49	19.92	236	107.94	19.72
247	0	17.61	244	0	12.37	243	0.04	17.33	237	1.45	17.22
248	21.11	15.21	245	17.05	11.54	244	23.13	14.61	238	24.34	14.57
249	0	14.14	246	0	14.13	245	0	14.21	239	0	14.39
250	0	15.88	247	0	16.1	246	0	16.14	240	0.67	16.71
251	0.01	17.16	248	0.17	17.8	247	1.27	17.2	241	0.5	17.82
252	0	18.48	249	0.34	18.98	248	0	18.79	242	0	18.84
253	0	19.26	250	0	22.54	249	0	19.24	243	0	19.29
254	1.78	22.54	251	1.71	23.14	250	0.2	22.54	244	0.67	22.42
255	58.17	22.84	252	55.59	26.97	251	15.1	22.47	245	39.75	22.07
256	1.04	26.09	253	49.88	29.59	252	66.22	26.25	246	48.88	25.75
257	13.02	29.41	254	0.33	33.37	253	0	29.2	247	0	28.84
258	4.06	32.32	255	6.89	36.25	254	5.4	32.86	248	2.31	32.49
259	36.23	35.51	256	1	38.97	255	20.95	35.64	249	6.89	35.46
260	28.57	38.17	257	6.32	42.72	256	12.32	38.74	250	35.55	38.43
261	106.53	41.85	258	38.78	44.77	257	41.04	42.11	251	28.54	41.89
262	138.2	45.11	259	101.59	47.83	258	137.66	44.1	252	53.2	45.54
-	-	-	-	-	-	259	114.23	46.96	-	-	-
263	54.88	47.44	260	22.43	50.19	260	19.11	48.19	253	152.19	47.47
264	69.24	49.33	261	112.1	51.83	261	119.35	50.32	254	16.11	48.23
-	-	-	262	65.41	48.87	-	-	-	-	-	-
265	8.09	46.51	263	26.39	47.01	262	15.75	48.38	255	33.23	50.61
266	7.66	45.97	264	11.34	45.28	263	23.49	47.76	256	34.44	47.81
-	-	-	-	-	-	-	-	-	257	95.11	48.35
267	15.2	44.75	265	37.43	47.4	264	22.45	45.5	258	46.58	46.14
268	8.06	46.96	266	9.2	47.18	265	1.95	48.02	259	13.22	48.38
269	119.64	46.51	267	85.04	49.43	266	96.88	47.65	260	145.5	47.31
270	9.08	48.97	268	7.92	49.82	267	7.01	48.74	261	26.07	49.56

271	86.95	49.42	269	98.73	52.59	268	142.17	48.28	262	102.92	49.83
272	10.63	52.36	270	19.62	52.34	269	63.65	50.14	263	37.83	52.86
273	73.9	52.44	271	73.81	51.54	270	140.07	48.93	264	110.36	53.26
274	53.84	52.2	272	44.3	54.28	271	63	49.6	265	55.55	52.28
275	48.76	55.19	273	86	55.48	272	24.87	53.26	266	28.45	54.62
276	109.33	56.86	274	125.23	57.8	273	126.83	53.97	267	155.74	55.32
277	44.22	59.44	275	31.42	58.24	274	31.62	56.59	268	26.23	57.6
278	131.06	59.76	276	99.71	59.32	275	90.54	57.4	269	105.93	58.09
279	17.73	59.73	277	15.16	59.87	276	14.68	58.78	270	11.25	59.71
280	71.16	58.89	278	140.93	58.92	277	138.32	59.45	271	63.46	60.85
281	0.28	58.33	279	4.18	56.18	278	2.68	58.74	272	0	59.92
282	0.17	55.44	280	0.43	55.56	279	0	56.15	273	0	57.06
283	0	55.39	281	1.17	53.38	280	0	55.66	274	0.17	56.71
284	22.07	52.91	282	14.22	56.46	281	12.67	53	275	52	54.52
285	69.62	56.02	283	98.23	57.33	282	90.21	55.76	276	112.82	57.55
286	0	56.36	284	0	58.74	283	0	56.94	277	0	58.34
287	0.27	58.23	285	4.66	62.02	284	4.87	58.62	278	0.74	59.51
288	1.39	61.58	286	3.6	64.8	285	6.43	61.89	279	8.54	62.93
289	54.85	64.35	287	147.14	67.57	286	147.16	64.64	280	86.8	65.19
290	46.16	64.79	288	42.05	69.45	287	45.03	67.68	281	28.03	68.35
291	78.63	68.48	289	113	70.42	288	115.28	69.96	282	142.12	70.72
292	44.6	69.38	290	51.46	69.54	289	48.86	70.38	283	40.33	71.81
293	14.71	68.6	291	61.75	67.87	290	20	69.95	284	61.64	70.93
294	8.34	67.04	292	63.17	65.75	291	2.34	68.09	285	49.97	69.45
295	3.56	64.87	293	1.16	64.77	292	0.94	66.14	286	1.46	67.36
296	36.48	64.13	294	49.1	62.02	293	36.18	64.99	287	37.07	66.48
297	25.66	61.46	295	20.73	58.85	294	18.18	61.95	288	5.71	63.41
298	26.91	58.3	296	34.09	57.78	295	41.21	59.01	289	8.04	60.69
299	56.94	57.33	297	84.08	54.6	296	43.03	58.45	290	136.35	60.13
300	35.78	54.14	298	64.11	55.51	297	44.94	55.19	291	132.93	56.59
301	19.29	54.93	299	57.75	54.23	298	40.16	55.84	292	17.33	57.98
302	2.03	53.5	300	12.55	56.64	299	21.03	54.65	293	5.86	56.51
303	2.43	55.88	301	44.39	60.27	300	44.34	57.06	294	40.51	57.26
304	7.51	59.63	302	130.9	61.71	301	149.57	60.84	295	104.4	60.89
305	9.07	60.72	303	38.64	64.61	302	34.13	61.89	296	47.35	62.64
306	3.52	63.7	304	18.43	64.29	303	17	64.91	297	24.92	65.97
307	55.21	63.42	305	143.58	62.88	304	29.53	64.92	298	133.79	66.07
308	54.45	62.46	306	82.78	65.41	305	35.7	63.69	299	84.49	65.03
309	0	65.31	307	41.72	66.16	306	0.08	66.1	300	35.33	67.86

310	106.23	65.94	308	227.31	69.71	307	92.32	66.42	301	62.55	67.8
311	38.51	69.26	309	40.02	71.06	308	4.2	69.94	302	54.41	71.18
312	96.69	69.4	310	124.44	74.05	309	132.78	70.82	303	178.61	72.68
313	67.93	72.71	311	95.27	75.08	310	105.73	73.76	304	56.5	75.13
314	6.17	74.51	312	55.62	78.34	311	5.84	75.03	305	56.42	76.46
315	69.58	77.57	313	78.31	80.47	312	10.47	78.22	306	65.96	79.79
316	1.69	79.02	314	104.65	84.2	313	1.82	80.79	307	97.38	81.62
317	0.64	82.8	315	44.95	85.8	314	0.81	84.53	308	51.43	85.38
318	14.98	83.81	316	85.45	89.08	315	25.99	85.71	309	100.51	86.59
319	0	87.52	317	24.93	91.6	316	0	89.04	310	25.09	90.06
320	0	90.52	318	73.3	92.41	317	0.37	91.67	311	70.26	92.76
321	70.82	91.51	319	133.03	93.22	318	69.78	92.31	312	115.16	93.61
322	3.33	92.62	320	12.82	96.59	319	14.87	93.11	313	13.67	94.56
323	23.53	96.18	-	-	-	-	-	-	314	60.16	98
324	36.87	97.29	321	87.78	97.67	320	19.88	96.28	315	99.16	99.73
325	84.47	100.73	322	76.34	100.8	-	-	-	316	111.86	103.46
326	120.01	100.54	323	87.54	101.59	-	-	-	317	140.95	104.24
327	145.66	97.04	-	-	-	321	176.12	97.51	-	-	-
328	138.18	97.13	-	-	-	-	-	-	-	-	-

Table S7. The sources for antigenic data (HI data) used in this study. The Archetti-Horsfall distance (dAH) between viral strains of influenza A(H1N1), A(H3N2) and A(H5N1) virus could be derived from them with the method described above.

Part I Sources for HI data of influenza A(H1N1) virus

1 Ndifon W, Dushoff J, Levin S A. On the use of hemagglutination-inhibition for influenza surveillance: surveillance data are predictive of influenza vaccine effectiveness. Vaccine, 2009, 27(18): 2447-2452.

2 Daniels R S, Douglas A R, Skehel J J, et al. Antigenic and amino acid sequence analyses of influenza viruses of the H1N1 subtype isolated between 1982 and 1984[J]. Bulletin of the World Health Organization, 1985, 63(2): 273.

3 Nakajima S, Cox N J, Kendal A P. Antigenic and genomic analyses of influenza A (H1N1) viruses from different regions of the world, February 1978 to March 1980[J]. Infection and immunity, 1981, 32(1): 287-294.

4 Kendal A P, Joseph J M, Kobayashi G, et al. LABORATORY-BASED SURVEILLANCE OF INFLUENZA VIRUS IN THE UNITED STATES DURING THE WINTER OF 1977–1978 I PERIODS OF PREVALENCE OF H1N1 AND H3N2 INFLUENZA A STRAINS, THEIR RELATIVE RATES OF ISOLATION IN DIFFERENT AGE GROUPS, AND DETECTION OF ANTIGENIC VARIANTS[J]. American journal of epidemiology, 1979, 110(4): 449-461.
5 Cox N J, Bai Z S, Kendal A P. Laboratory-based surveillance of influenza A (H1N1) and A (H3N2) viruses in 1980-81: antigenic and genomic analyses[J]. Bulletin of the World Health Organization, 1983, 61(1): 143.
6 Centers for Disease Control and Prevention, American, 2009. Information for the vaccines and related biological products advisory committee
7 Centers for Disease Control and Prevention 2004. Information for the vaccines and related biological products advisory committee
8 WHO Collaborating Center For Reference & Research On Influenza. Annual Report, Australia, 2006
9 Hay A J, Lin Y P, Gregory V and Bennett M. WHO INFLUENZA CENTRE LONDON, ANNUAL REPORT, August 2001 to July 2002
10 Hay A J, Lin Y P, Gregory V and Bennett M. WHO INFLUENZA CENTRE LONDON, ANNUAL REPORT, August 2002 to August 2003
11 Hay A J, Lin Y P, Gregory V and Bennett M. WHO INFLUENZA CENTRE LONDON, ANNUAL REPORT,August 2003 to July 2004
12 Hay A J, Lin Y P, Gregory V and Bennett M.CHARACTERISTICS OF HUMAN INFLUENZA A H1N1, A H3N2 AND B VIRUSES ISOLATED OCTOBER 2004 TO JANUARY 2005. 2005, February.
13 Hay A J, Lin Y P, Gregory V and Bennett M. CHARACTERISTICS OF HUMAN INFLUENZA AH1N1, AH3N2 AND B VIRUSES ISOLATED FEBRUARY TO JULY 2005. 2005, September.
14 Hay A J, Lin Y P, Gregory V and Bennett M. CHARACTERISTICS OF HUMAN INFLUENZA AH1N1, AH3N2 AND B VIRUSES ISOLATED OCTOBER 2005 TO FEBRUARY 2006. 2006, March.
15 Hay A J, Lin Y P, Gregory V and Bennett M.CHARACTERISTICS OF HUMAN INFLUENZA AH1N1, AH3N2 AND B VIRUSES ISOLATED JANUARY TO SEPTEMBER 2006. 2006, September.
16 Hay, A J, Daniels R, Lin Y P, Zheng X, Gregory V, Bennett M and Whittaker L. CHARACTERISTICS OF HUMAN INFLUENZA AH1N1, AH3N2 AND B VIRUSES ISOLATED SEPTEMBER 2006 TO FEBRUARY 2007. 2007, March.
17 Hay, A J, Daniels R, Lin Y P, Zheng X, Gregory V, Bennett M and Whittaker L. CHARACTERISTICS OF HUMAN INFLUENZA AH1N1, AH3N2 AND B VIRUSES ISOLATED FEBRUARY TO AUGUST 2007. 2007, September.
18 Hay, A J, Daniels R, Lin Y P, Zheng X, Gregory V, Bennett M and Whittaker L. CHARACTERISTICS OF HUMAN INFLUENZA AH1N1, AH3N2, AND B VIRUSES ISOLATED SEPTEMBER 2007 TO FEBRUARY 2008. 2008, March.
19 Hay, A J, Daniels R, Lin Y P, Zheng X, Gregory V, Bennett M and Whittaker L. CHARACTERISTICS OF HUMAN INFLUENZA AH1N1, AH3N2, AND B VIRUSES ISOLATED FEBRUARY TO AUGUST 2008. 2008, September.

20 Hay, A J, Daniels R, Lin Y P, Zheng X, Hou T, Gregory V, Whittaker L, Kloess J and Cattle N. WHO INFLUENZA CENTRE, LONDON, 2009, February.
21 Hay, A J, Daniels R, Lin Y P, Zheng X, Hou T, Gregory V, Whittaker L, Kloess J and Cattle N. WHO INFLUENZA CENTRE, LONDON, 2009, September.
22 McCauley J, Daniels R, Lin Y P, Zheng X, Hou T, Gregory V, Whittaker L, Cattle N, Kloess J and Halai C. WHO INFLUENZA CENTRE, LONDON, 2010, February.
23 McCauley J, Daniels R, Lin Y P, Zheng X, Gregory V, Whittaker L, Cattle N, Halai C, Cross K and Kloess J. WHO INFLUENZA CENTRE, LONDON, 2011, February.
24 McCauley J, Daniels R, Lin Y P, Zheng X, Gregory V, Whittaker L, Cattle N, Halai C and Cross K. WHO INFLUENZA CENTRE, LONDON, 2012, February.

Part II Sources for HI data of influenza A(H3N2) virus

1 Ndifon W, Dushoff J, Levin S A. On the use of hemagglutination-inhibition for influenza surveillance: surveillance data are predictive of influenza vaccine effectiveness. Vaccine, 2009, 27(18): 2447-2452.
2 Cox N J, Bai Z S, Kendal A P. Laboratory-based surveillance of influenza A (H1N1) and A (H3N2) viruses in 1980-81: antigenic and genomic analyses. Bulletin of the World Health Organization, 1983, 61(1): 143.
3 Kendal A P, Joseph J M, Kobayashi G, et al. LABORATORY-BASED SURVEILLANCE OF INFLUENZA VIRUS IN THE UNITED STATES DURING THE WINTER OF 1977–1978 I PERIODS OF PREVALENCE OF H1N1 AND H3N2 INFLUENZA A STRAINS, THEIR RELATIVE RATES OF ISOLATION IN DIFFERENT AGE GROUPS, AND DETECTION OF ANTIGENIC VARIANTS[J]. American journal of epidemiology, 1979, 110(4): 449-461.
4 Both G W, Sleigh M J, Cox N J, et al. Antigenic drift in influenza virus H3 hemagglutinin from 1968 to 1980: multiple evolutionary pathways and sequential amino acid changes at key antigenic sites[J]. Journal of virology, 1983, 48(1): 52-60.
5 Besselaar T G, Botha L, McAnerney J M, et al. Antigenic and molecular analysis of influenza A (H3N2) virus strains isolated from a localised influenza outbreak in South Africa in 2003[J]. Journal of medical virology, 2004, 73(1): 71-78.
6 Bulimo W D, Garner J L, Schnabel D C, et al. Genetic analysis of H3N2 influenza A viruses isolated in 2006–2007 in Nairobi, Kenya[J]. Influenza and other respiratory viruses, 2008, 2(3): 107-113.
7 Coiras M T, Aguilar J C, Galiano M, et al. Rapid molecular analysis of the haemagglutinin gene of human influenza A H3N2 viruses isolated in Spain from 1996 to 2000[J]. Archives of virology, 2001, 146(11): 2133-2147.
8 Daum LT, Shaw MW, Klimov AI, et al. Influenza A (H3N2) Outbreak, Nepal. Emerging Infectious Diseases. 2005;11(8):1186-1191.
9 Ellis J S, Chakraverty P, Clewley J P. Genetic and antigenic variation in the haemagglutinin of recently circulating human influenza A (H3N2) viruses in the United Kingdom[J]. Archives of virology, 1995, 140(11): 1889-1904.
10 Ellis J S, Sadler C J, Laidler P, et al. Analysis of influenza A H3N2 strains isolated in England during 1995–1996 using polymerase chain reaction restriction[J]. Journal of medical virology, 1997, 51(3): 234-241.

11 Gregory V, Lim W, Cameron K, et al. Infection of a child in Hong Kong by an influenza A H3N2 virus closely related to viruses circulating in European pigs[J]. Journal of General Virology, 2001, 82(6): 1397-1406.
12 Pechirra P, Gonçalves P, Arraiolos A, et al. Characterization of influenza A/Fujian/411/2002 (H3N2) ‐ like viruses isolated in Portugal between 2003 and 2005[J]. Journal of medical virology, 2008, 80(9): 1624-1630.
13 Centers for Disease Control and Prevention (CDC. Update: influenza activity--United States and worldwide, 1994-95 season, and composition of the 1995-96 influenza vaccine[J]. MMWR. Morbidity and mortality weekly report, 1995, 44(15): 292.
14 Centers for Disease Control and Prevention (CDC. Update: influenza activity--United States and Worldwide, 1995-96 season, and composition of the 1996-97 influenza vaccine[J]. MMWR. Morbidity and mortality weekly report, 1996, 45(16): 326.
15 Update C D C. influenza activity—United States, 1997–98 season[J]. MMWR, 1997, 46: 1094-8.
16 World Health Organization. Influenza. Weekly Epidemiological Record, 1972.
17 World Health Organization. Recommended composition of influenza virus vaccines for use in the 1988-1989 season. Weekly Epidemiological Record, 26th February 1988.
18 WHO Collaborating Center For Reference & Research On Influenza. Annual Report, Australia, 2006
19 Hay A J, Lin Y P, Gregory V and Bennett M. WHO INFLUENZA CENTRE LONDON, ANNUAL REPORT, August 2001 to July 2002
20 Hay A J, Lin Y P, Gregory V and Bennett M. WHO INFLUENZA CENTRE LONDON, ANNUAL REPORT, August 2002 to August 2003
21 Hay A J, Lin Y P, Gregory V and Bennett M. WHO INFLUENZA CENTRE LONDON, ANNUAL REPORT,August 2003 to July 2004
22 Hay A J, Lin Y P, Gregory V and Bennett M.CHARACTERISTICS OF HUMAN INFLUENZA A H1N1, A H3N2 AND B VIRUSES ISOLATED OCTOBER 2004 TO JANUARY 2005. 2005, February.
23 Hay A J, Lin Y P, Gregory V and Bennett M. CHARACTERISTICS OF HUMAN INFLUENZA AH1N1, AH3N2 AND B VIRUSES ISOLATED FEBRUARY TO JULY 2005. 2005, September.
24 Hay A J, Lin Y P, Gregory V and Bennett M. CHARACTERISTICS OF HUMAN INFLUENZA AH1N1, AH3N2 AND B VIRUSES ISOLATED OCTOBER 2005 TO FEBRUARY 2006. 2006, March.
25 Hay A J, Lin Y P, Gregory V and Bennett M.CHARACTERISTICS OF HUMAN INFLUENZA AH1N1, AH3N2 AND B VIRUSES ISOLATED JANUARY TO SEPTEMBER 2006. 2006, September.
26 Hay, A J, Daniels R, Lin Y P, Zheng X, Gregory V, Bennett M and Whittaker L. CHARACTERISTICS OF HUMAN INFLUENZA AH1N1, AH3N2 AND B VIRUSES ISOLATED SEPTEMBER 2006 TO FEBRUARY 2007. 2007, March.
27 Hay, A J, Daniels R, Lin Y P, Zheng X, Gregory V, Bennett M and Whittaker L. CHARACTERISTICS OF HUMAN INFLUENZA AH1N1, AH3N2 AND B VIRUSES ISOLATED FEBRUARY TO AUGUST 2007. 2007, September.
28 Hay, A J, Daniels R, Lin Y P, Zheng X, Gregory V, Bennett M and Whittaker L.

CHARACTERISTICS OF HUMAN INFLUENZA AH1N1, AH3N2, AND B VIRUSES ISOLATED SEPTEMBER 2007 TO FEBRUARY 2008. 2008, March.
29 Hay, A J, Daniels R, Lin Y P, Zheng X, Gregory V, Bennett M and Whittaker L. CHARACTERISTICS OF HUMAN INFLUENZA AH1N1, AH3N2, AND B VIRUSES ISOLATED FEBRUARY TO AUGUST 2008. 2008, September.
30 Hay, A J, Daniels R, Lin Y P, Zheng X, Hou T, Gregory V, Whittaker L, Kloess J and Cattle N. WHO INFLUENZA CENTRE, LONDON, 2009, February.
31 Hay, A J, Daniels R, Lin Y P, Zheng X, Hou T, Gregory V, Whittaker L, Kloess J and Cattle N. WHO INFLUENZA CENTRE, LONDON, 2009, September.
32 McCauley J, Daniels R, Lin Y P, Zheng X, Hou T, Gregory V, Whittaker L, Cattle N, Kloess J and Halai C. WHO INFLUENZA CENTRE, LONDON, 2010, February.
33 McCauley J, Daniels R, Lin Y P, Zheng X, Gregory V, Whittaker L, Cattle N, Halai C, Cross K and Kloess J. WHO INFLUENZA CENTRE, LONDON, 2011, February.
34 McCauley J, Daniels R, Lin Y P, Zheng X, Gregory V, Whittaker L, Cattle N, Halai C and Cross K. WHO INFLUENZA CENTRE, LONDON, 2012, February.
35 Spencer, J; Hampson, A; Yohannes, K; Roche, P and Miller, M. Annual report of the National Influenza Surveillance Scheme, 2003. Communicable Diseases Intelligence Quarterly Report, 2004, 28(2): 160-8.
36 Spencer, JD; Roche, PW; Yohannes, K; Hampson, A and Li, J. Annual report of the National Influenza Surveillance Scheme, 2004. Communicable Diseases Intelligence Quarterly Report, 2005, 29(2): 125-36
37 Walker, JC; Barr, IG; Roche, PW and Firestone, SM. Annual report of the National Influenza Surveillance Scheme, 2005. Communicable Diseases Intelligence Quarterly Report,2006,30(2):189-200
38 Barr, IG and O'Brien, K. Annual report of the National Influenza Surveillance Scheme, 2006. Communicable Diseases Intelligence Quarterly Report, 2007, 31(2): 167-79.
39 Kaczmarek, M; Pengilley, A; Paterson, B; Owen, R; Liu, C and Barr, IG. Annual report of the National Influenza Surveillance Scheme, 2007. Communicable Diseases Intelligence Quarterly Report, 2007, 32(2):208-26

Part III Sources for HI data of influenza A(H5N1) virus

1 Lindsay E. Edwards, Doan C. Nguyen, Xiuhua Lu, Henrietta Hall, Amanda Balish, Jan E. Mabry, Wilina Lim, Nancy J.Cox, Alexander Klimov, Jacquelin M. Katz. Antigenic characteristics of recent avian influenza A H5N1 viruses isolated from humans. International Congress Series 1263, 2004, 109 – 113
2 World Health Organization. Antigenic and genetic characteristics of H5N1 viruses and candidate H5N1 vaccine viruses developed for potential use as pre-pandemic vaccines. 2006.
3 The World Health Organization Global Influenza Program Surveillance Network. Evolution of H5N1 Avian Influenza Viruses in Asia . Emerging Infectious Diseases, 2005, 11(10).
4 Wai Lan Wu, Yixin Chen, Pui Wang, Wenjun Song, Siu-Ying Lau, Jane M., Rayner, Gavin J. D. Smith, Robert G. Webster, J. S. Malik Peiris, Tianwei Lin, Ningshao Xia, Yi Guan and Honglin Chen. Antigenic profile of avian H5N1 viruses in Asia from 2002-2007 . J Virol, 2008, (82):1798-807.
5 World Health Organization. Antigenic and genetic characteristics of H5N1 viruses and

candidate H5N1 vaccine viruses developed for potential use as human vaccines. 2008, February
6 World Health Organization. Antigenic and genetic characteristics of H5N1 viruses and candidate H5N1 vaccine viruses developed for potential use as human vaccines. 2008, September
7 World Health Organization. Antigenic and genetic characteristics of H5N1 viruses and candidate H5N1 vaccine viruses developed for potential use as human vaccines. 2009, February
8 World Health Organization. Antigenic and genetic characteristics of H5N1 viruses and candidate H5N1 vaccine viruses developed for potential use as human vaccines. 2009, September
9 World Health Organization. Antigenic and genetic characteristics of H5N1 viruses and candidate H5N1 vaccine viruses developed for potential use as human vaccines. 2010, February
10 World Health Organization. Antigenic and genetic characteristics of H5N1 viruses and candidate H5N1 vaccine viruses developed for potential use as human vaccines. 2010, September
11 World Health Organization. Antigenic and genetic characteristics of H5N1 viruses and candidate H5N1 vaccine viruses developed for potential use as human vaccines. 2011, February
12 Katharine M. Sturm-Ramirez, Trevor Ellis, Barry Bousfield, Yi Guan, Malik Peiris, Robert Webster. H5N1 influenza A viruses from 2002 are highly pathogenic in waterfowl. International Congress Series, 2004, 1263:200 – 204.
13 Katharine M. Sturm-Ramirez, Trevor Ellis, Barry Bousfield, Lucy Bissett,Kitman Dyrting, Jerold E. Rehg, Leo Poon, Yi Guan,Malik Peiris, and Robert G. Webster. Reemerging H5N1 Influenza Viruses in Hong Kong in 2002 Are Highly Pathogenic to Ducks. J Virol, 2004, 4892–4901
14 G. J. D. Smith, X. H. Fan, J. Wang, K. S. Li, K. Qin, J. X. Zhang, D. Vijaykrishna, C. L. Cheung, K. Huang, J. M. Rayner, J. S. M. Peiris, H. Chen, R. G. Webster, and Y. Guan. Emergence and predominance of an H5N1 influenza variant in China. PNAS, 2006, 16936–16941.
15 M. F. Ducatez, C. M. Olinger, A. A. Owoade, Z. Tarnagda, M. C. Tahita, A. Sow, S. De Landtsheer, W. Ammerlaan, J. B. Ouedraogo, A. D. M. E. Osterhaus, R. A. M. Fouchier and C. P. Muller. Molecular and antigenic evolution and geographical spread of H5N1 highly pathogenic avian influenza viruses in western Africa. Journal of General Virology, 2007, 88:2297–2306.
16 Tung Nguyen, C. Todd Davis, William Stembridge, Bo Shu, Amanda Balish, Kenjiro Inui, Hoa T. Do, Huong T. Ngo, Xiu-Feng Wan, Margaret McCarron, Stephen E. Lindstrom, Nancy J. Cox, Cam V. Nguyen, Alexander I. Klimov, Ruben O. Donis. Characterization of a highly pathogenic avian influenza H5N1 virus sublineage in poultry seized at ports of entry into Vietnam. Virology, 2009, 387:250–256.
17 Jie Dong, Yumiko Matsuoka, Taronna R. Maines, David E. Swayne, Eduardo O'Neill, C. Todd Davis, Neal Van-Hoven, Amanda Balish, Hong-jie Yu, Jacqueline M. Katz, Alexander Klimov, Nancy Cox, De-xin Li, Yu Wang, Yuan-ji Guo, Wei-zhong Yang, Ruben O. Donis, Yue-long Shu. Development of a new candidate H5N1 avian influenza virus for pre-pandemic vaccine production. Influenza and Other Respiratory Viruses, 2009, 3(6), 287–295.
18 Yanbing Li, Jianzhong Shi, Gongxun Zhong, Guohua Deng, Guobin Tian, Jinying Ge, Xianying Zeng, Jiasheng Song, Dongming Zhao, Liling Liu, Yongping Jiang, Yuntao Guan, Zhigao Bu, and Hualan Chen. Continued Evolution of H5N1 Influenza Viruses in Wild Birds, Domestic Poultry, and Humans in China from 2004 to 2009. J Virol, 2010, 8389–8397.
19 Amanda L. Balish, C. Todd Davis, Magdi D. Saad, Nasr El-Sayed, Hala Esmat, Jeffrey A. Tjaden, Kenneth C. Earhart, Lu'ay E. Ahmed, Mohamed Abd El-Halem, Abdel Hakem M. Ali, Samir A. Nassif, Elham A. El-Ebiary, M. Taha, Mona M. Aly, Abdelstattar Arafa, Eduardo

O'Neill, Xu Xiyan, Nancy J. Cox, Ruben O. Donis, and Alexander I. Klimov. Antigenic and Genetic Diversity of Highly Pathogenic Avian Influenza A (H5N1) Viruses Isolated in Egypt. AVIAN DISEASES, 2010, 54:329–334.
20 World Health Organization. Antigenic and genetic characteristics of H5N1 viruses and candidate H5N1 vaccine viruses developed for potential use as human vaccines. 2011, September
21 World Health Organization. Antigenic and genetic characteristics of zoonotic influenza viruses and development of candidate vaccine viruses for pandemic preparedness. 2011,September

Part IV Sources for HI data of influenza A(H9N2) virus

1 WHO. Antigenic and genetic characteristics of influenza A(H5N1) and influenza A(H9N2) viruses and candidate vaccine viruses developed for potential use in human vaccines. February, 2010.
2 Shanmuganatham K, Feeroz M M, Jones-Engel L, et al. Antigenic and molecular characterization of avian influenza A (H9N2) viruses, Bangladesh. Emerging infectious diseases, 2013, 19(9): 1393.
3 Cong Y L, Pu J, Liu Q F, et al. Antigenic and genetic characterization of H9N2 swine influenza viruses in China. Journal of General Virology, 2007, 88(7): 2035-2041.
4 Sun Y, Pu J, Jiang Z, et al. Genotypic evolution and antigenic drift of H9N2 influenza viruses in China from 1994 to 2008. Veterinary microbiology, 2010, 146(3): 215-225.
5 Zhang Y, Yin Y, Bi Y, et al. Molecular and antigenic characterization of H9N2 avian influenza virus isolates from chicken flocks between 1998 and 2007 in China. Veterinary microbiology, 2012, 156(3): 285-293.
6 Zhu Y, Hu S, Bai T, et al. Phylogenetic and antigenic characterization of reassortant H9N2 avian influenza viruses isolated from wild waterfowl in the East Dongting Lake wetland in 2011–2012. Virol J, 2014, 11(1): 77.

Supplementary References

1 Ndifon, W., Dushoff, J. & Levin, S. A. On the use of hemagglutination-inhibition for influenza surveillance: Surveillance data are predictive of influenza vaccine effectiveness. *Vaccine* **27**, 2447-2452, doi:DOI 10.1016/j.vaccine.2009.02.047 (2009).
2 Liao, Y. C., Lee, M. S., Ko, C. Y. & Hsiung, C. A. Bioinformatics models for predicting antigenic variants of influenza A/H3N2 virus. *Bioinformatics* **24**, 505-512, doi:DOI 10.1093/bioinformatics/btm638 (2008).
3 Bernstein, F. C. *et al.* The Protein Data Bank: a computer-based archival file for macromolecular structures. *Archives of biochemistry and biophysics* **185**, 584-591 (1978).
4 Zhang, Y. & Skolnick, J. TM-align: a protein structure alignment algorithm based on the TM-score. *Nucleic Acids Res* **33**, 2302-2309, doi:Doi 10.1093/Nar/Gki524 (2005).
5 Sayle R, B. A. in *Proceedings of the 10th Eurographics UK.*
6 Bush, R. M., Bender, C. A., Subbarao, K., Cox, N. J. & Fitch, W. M. Predicting the evolution of human influenza A. *Science* **286**, 1921-1925 (1999).

7 Bush, R. M., Fitch, W. M., Bender, C. A. & Cox, N. J. Positive selection on the H3 hemagglutinin gene of human influenza virus A. *Molecular biology and evolution* **16**, 1457-1465 (1999).

8 Lees, W. D., Moss, D. S. & Shepherd, A. J. A computational analysis of the antigenic properties of haemagglutinin in influenza A H3N2. *Bioinformatics* **26**, 1403-1408, doi:10.1093/bioinformatics/btq160 (2010).
